# Successful Surveillance Using Endometrial Cytology in a Woman With Lynch Syndrome

**DOI:** 10.7759/cureus.66250

**Published:** 2024-08-06

**Authors:** Atsushi Murakami, Hidetaka Nomura, Yuko Sugiyama, Hiroyuki Kanao

**Affiliations:** 1 Department of Gynecology, Cancer Institute Hospital of Japanese Foundation for Cancer Research (JFCR), Tokyo, JPN

**Keywords:** lynch syndrome, hereditary tumor, endometrial cytology, surveillance, endometrial cancer (ec)

## Abstract

Lynch syndrome (LS) results from pathogenic variants in mismatch repair genes and is the most common hereditary cancer syndrome. Some guidelines or studies recommend restricting screening according to endometrial cancer (EC) using endometrial biopsy. The pooled sensitivity and specificity of endometrial cytology for detecting endometrial atypical hyperplasia or cancer have been reported to be as high as the pooled sensitivity and specificity of endometrial biopsy. We conduct transvaginal ultrasound and endometrial cytology in women with LS every six months as surveillance for gynecological malignancy. Through this surveillance program, we can detect early-stage EC in women with LS. Here, we report the case of a patient with stage IA EC detected by endometrial cytology and treated completely. The patient was a 47-year-old woman under surveillance for gynecological malignancy. She was diagnosed as having LS with a germline pathogenic variant in *MSH6* after surgery for rectal cancer. Thereafter, gynecological surveillance was started. She had regular menstruation and never experienced atypical genital bleeding. However, her cytopathological findings indicated grade 1 endometrial carcinoma. Endometrial biopsy was performed and endometrial carcinoma was confirmed pathologically. A laparoscopic modified radical hysterectomy with bilateral salpingo-oophorectomy was performed. The resected specimen was reviewed pathologically, and the tumor was finally diagnosed as grade 1 endometrioid carcinoma confined to the endometrium without lymphovascular space invasion. She has remained asymptomatic and free of cancer for five years without any adjuvant therapy. We achieved successful surveillance using endometrial cytology. Endometrial cytology could replace endometrial biopsy as a screening tool for EC.

## Introduction

Lynch syndrome (LS) results from pathogenic variants in mismatch repair genes and is the most common hereditary cancer syndrome. According to data from the United States, 1 in 440 individuals has LS, and according to a study from Denmark, 1 in 278 individuals has LS [1]. For a woman with LS, the lifetime risks of endometrial and ovarian cancers are 40-60% and 10-17%, respectively, and the incidence increases with age beyond 40 years [1]. Currently, the screening criteria for LS-associated endometrial cancer (EC) are controversial. Some guidelines or studies recommend restricting screening according to risk factors, including age and family history [2-5]. Surveillance for EC aims to detect premalignant disease or early-stage cancer to increase survival, and it can be performed by symptom review, transvaginal ultrasound, blind endometrial biopsy, or hysteroscopy with targeted endometrial biopsy.

Since 1987, direct endometrial cytology has been the most common method of EC screening in Japan [6]. Although the most promising method for detecting EC is endometrial biopsy, a major barrier to its successful use among outpatients is the discomfort it causes. By contrast, direct endometrial cytology is relatively simple to perform and causes much less pain. It is currently the most common test for the initial evaluation of EC in Japan [7], and its use as the first-level screening method for women at high risk for EC has been encouraged [8]. The pooled sensitivity and specificity of endometrial cytology for detecting endometrial atypical hyperplasia (AH) or cancer have been reported to be as high as the pooled sensitivity and specificity of endometrial biopsy [9]. However, the effectiveness of endometrial cytology in asymptomatic women with a high risk of EC has not been validated so far.

We conduct transvaginal ultrasound and endometrial cytology in women with LS every six months as surveillance for gynecological malignancy. Through this surveillance program, we can detect early-stage EC in women with LS. Here, we report the case of a patient with stage IA EC detected by endometrial cytology and treated completely. To our knowledge, this is the first case report of a patient with LS detected with early-stage EC through a surveillance program using endometrial cytology. We suggest the inclusion of endometrial cytology instead of endometrial biopsy in surveillance programs for women with LS.

## Case presentation

**Figure 1:**
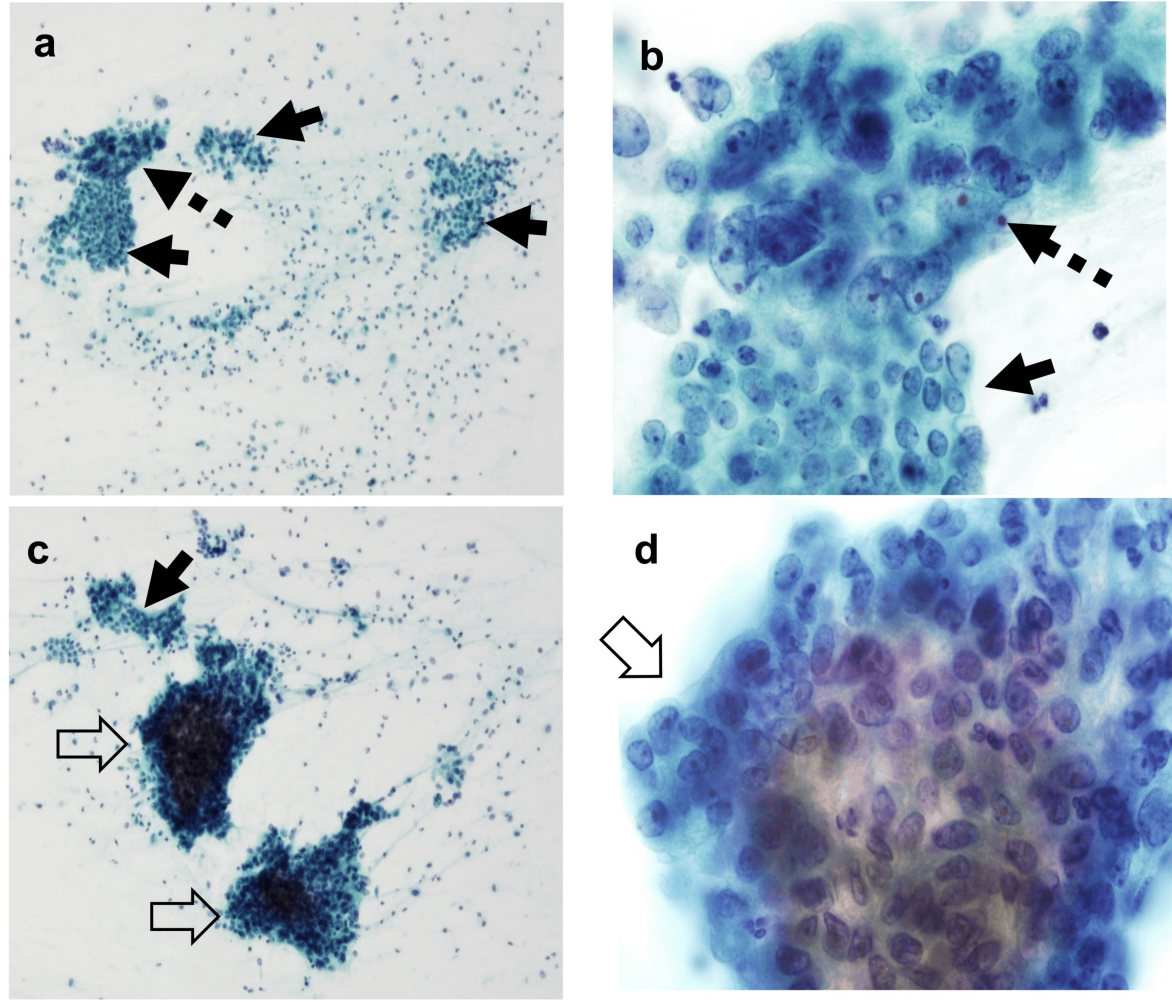
Cytological findings of the endometrial carcinoma (direct endometrial cytology using an endometrial brush). Normal proliferative endometrial cells (solid arrow), atypical endometrial cells (dot arrow), and grade 1 endometrioid carcinoma cells (open arrow) are seen (a-d). Lymphocytes are seen in the background (a, c). Papanicolaou stain; original magnification: ×10 (a, c), ×100 (b, d).

The patient was a 47-year-old woman under surveillance for gynecological malignancy. She had a history of rectal cancer at 38 years of age. Her father had experienced colon cancer and gastric cancer when he was 38 and 48 years old, respectively. She was diagnosed as having LS with a germline pathogenic variant in *MSH6* after surgery for rectal cancer. Thereafter, gynecological surveillance was started. She underwent cervical and endometrial cytology accompanied by transvaginal ultrasound every six months. She had regular menstruation and never experienced atypical genital bleeding. However, her cytopathological findings indicated grade 1 endometrial carcinoma (Figure 1).

**Figure 2:**
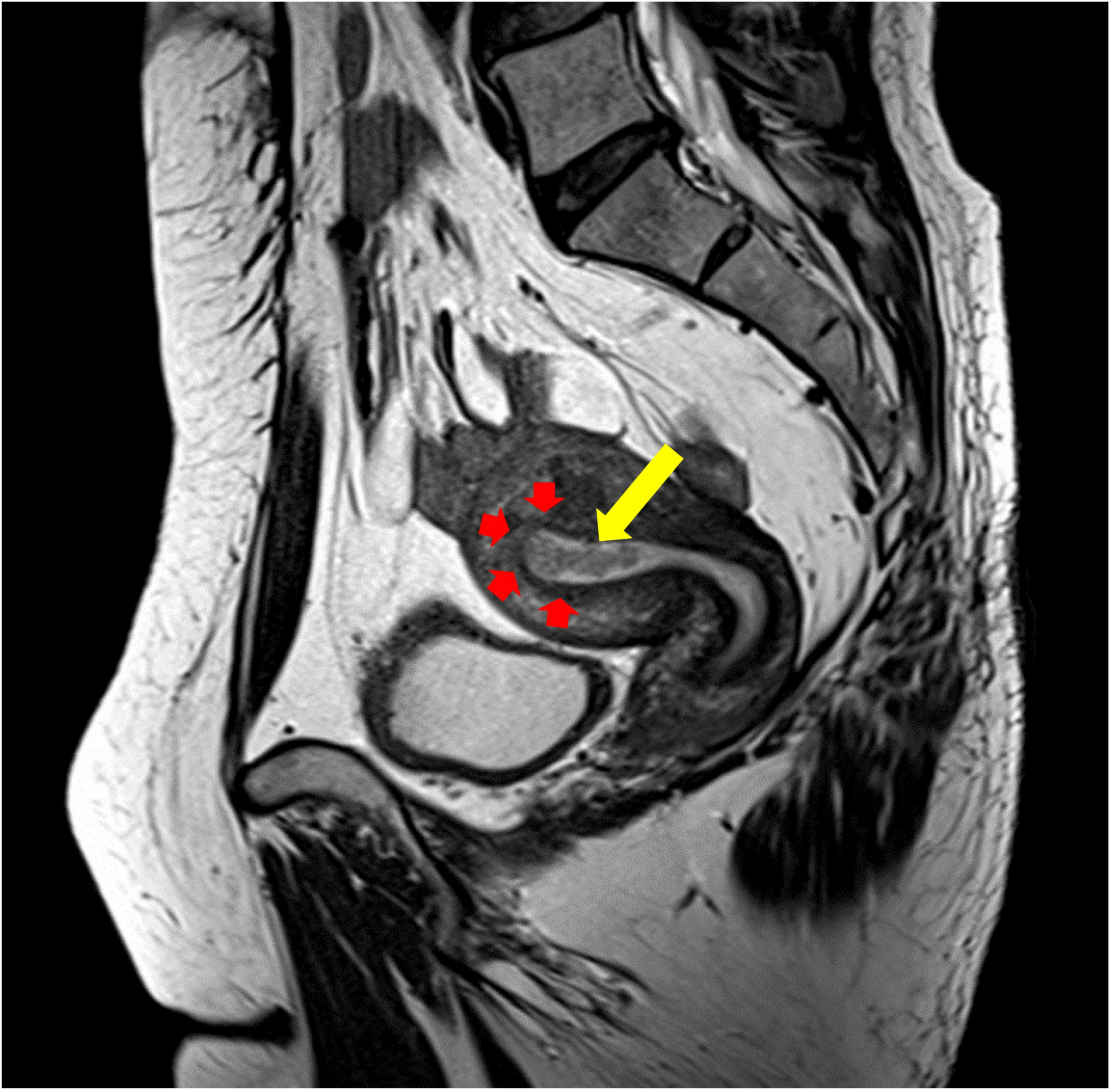
Pelvic magnetic resonance imaging (T2-weighted image). MRI revealed the presence of an endometrial tumor (yellow arrow) without myometrial invasion (red arrows).

At the time of the examination, endometrial thickness calculated by transvaginal ultrasound was 11 mm. Subsequently, endometrial biopsy was performed, and endometrial carcinoma grade 1 was confirmed. Serum levels of carbohydrate antigen (CA) 125, CA 19-9, and carcinoembryonic antigen were within the reference ranges. Pelvic magnetic resonance imaging (MRI) indicated a tumor in the uterine cavity with an intact junctional zone and a sharp tumor-myometrium interface (Figure 2).

**Figure 3:**
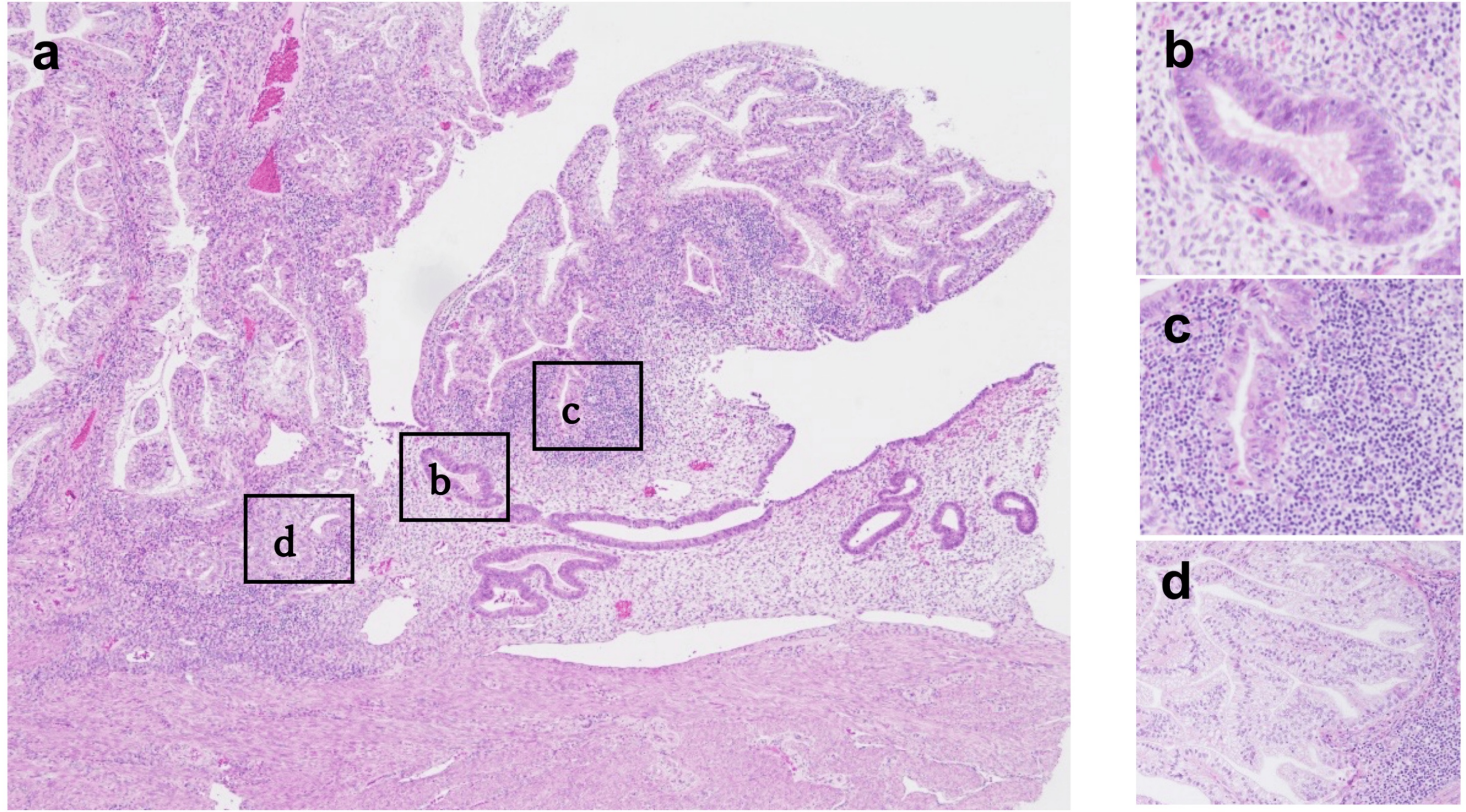
Histological findings of the endometrial carcinoma. Histological assessment shows an exophytic tumor diagnosed as an endometrial endometrioid carcinoma (a), with normal proliferative endometrial glands (b), atypical endometrial glands (c), and grade 1 endometrioid carcinoma cells (d). Marked tumor-infiltrating lymphocytes are seen (c, d). Hematoxylin and eosin stain; original magnification: ×10 (a), ×40 (b-d).

There was no lymph node metastasis or distant metastasis on 2-deoxy-2-[18F]fluoro-D-glucose positron emission tomography combined with computed tomography. A laparoscopic modified radical hysterectomy with bilateral salpingo-oophorectomy was performed. The resected specimen was reviewed pathologically, and the tumor was finally diagnosed as grade 1 endometrioid carcinoma confined to the endometrium without lymphovascular space invasion (Figure 3).

She has remained asymptomatic and free of cancer for five years without any adjuvant therapy.

## Discussion

The findings of this report indicate the effectiveness of endometrial cytology performed in asymptomatic women with LS for surveillance.

According to the National Comprehensive Cancer Network guidelines, EC screening does not have proven benefits in women with LS. However, endometrial biopsy is both highly sensitive and highly specific as a diagnostic procedure, and screening via endometrial biopsy every one to two years starting at the age of 30-35 years can be considered [5]. Similarly, according to the European Society for Medical Oncology guidelines, annual gynecological examination, transvaginal ultrasound with CA 125 analysis, and endometrial biopsy are recommended from the age of 30-35 years in women with LS [4]. Endometrial biopsy is considered the most dependable method in surveillance for LS.

Endometrial cytology has been reported to have relatively high sensitivity and specificity when compared with endometrial biopsy. A study conducted by Okadome et al. on 198 patients with EC, who underwent endometrial cytology, endometrial biopsy, and then hysterectomy, reported that for detecting type 1 endometrial carcinoma, the sensitivity and specificity of endometrial biopsy were 80% and 67%, respectively, while the sensitivity and specificity of endometrial cytology were 90.4% and 70.3%, respectively [10]. Moreover, for detecting type 2 endometrial carcinoma, the sensitivity and specificity of endometrial biopsy were 67.6% and 84.9%, respectively, while the sensitivity and specificity of endometrial cytology were 70.3% and 91.8%, respectively [10]. A study conducted by Tommaso et al. reported that for detecting endometrial AH or cancer, the sensitivity and specificity of endometrial biopsy were 93.7% and 100%, respectively, while the sensitivity and specificity of endometrial cytology were 87.5% and 63.8%, respectively, when malignancy, suspicious, or atypical scores were considered positive [11]. Both dilatation and curettage, as well as the gold standard of evaluating the endometrium (i.e., hysteroscopy-guided uterine biopsy), are painful and expensive and require anesthesia [12-14]. On the contrary, endometrial cytology is inexpensive, is tolerated well, and can be performed without anesthesia in an outpatient clinic. Since 1987, direct endometrial cytology has been the most common method of EC screening in Japan [6]. A recent meta-analysis showed that endometrial cytology has high diagnostic accuracy and could serve as a test to confirm or exclude endometrial AH or cancer. The pooled sensitivity and specificity of endometrial cytology for detecting endometrial AH or cancer were 0.91 (95% confidence interval (CI) = 0.74-0.97) and 0.96 (95% CI = 0.90-0.99), respectively [9]. Hence, endometrial cytology could replace endometrial biopsy as a screening tool for EC.

The effectiveness of gynecological surveillance in LS is unclear. Some studies have shown the benefit of gynecological surveillance in the early detection of EC, while others have shown no benefit [15]. Survival data are limited, and mortality data are lacking. However, both preinvasive (AH) and stage 1 diseases have been diagnosed in asymptomatic women undergoing EC surveillance [3]. Because of early detection, those who have preinvasive or stage 1 disease can preserve their fertility if they strongly desire. Moreover, the early detection of EC may result in minimally invasive therapy and the avoidance of adjuvant therapy. We believe that surveillance is effective in women with LS, even if it does not decrease the mortality rate. However, further research is needed to determine whether endometrial biopsy or cytology is more effective for surveillance in LS.

## Conclusions

We reported early detection of EC by endometrial cytology performed on asymptomatic women with LS for surveillance purposes. Endometrial cytology may be a minimally invasive approach with accuracy comparable to endometrial biopsy for surveillance of EC in women with LS. Early detection by surveillance is important to choose fertility-sparing treatment or to avoid highly invasive treatment. However, the effectiveness of surveillance in LS for EC requires further large-scale studies.
